# Transbilayer Phospholipid Movements in ABCA1-Deficient Cells

**DOI:** 10.1371/journal.pone.0000729

**Published:** 2007-08-15

**Authors:** Patrick Williamson, Margaret S. Halleck, Jonathan Malowitz, Susan Ng, Xiaoxuan Fan, Stephen Krahling, Alan T. Remaley, Robert A. Schlegel

**Affiliations:** 1 Department of Biology, Amherst College, Amherst, Massachusetts, United States of America; 2 Department of Biochemistry and Molecular Biology, The Pennsylvania State University, University Park, Pennsylvania, United States of America; 3 National Heart, Lung and Blood Institute, National Institutes of Health, Bethesda, Maryland, United States of America; University of Geneva, Switzerland

## Abstract

Tangier disease is an inherited disorder that results in a deficiency in circulating levels of HDL. Although the disease is known to be caused by mutations in the *ABCA1* gene, the mechanism by which lesions in the ABCA1 ATPase effect this outcome is not known. The inability of ABCA1 knockout mice (*ABCA1^−/−^*) to load cholesterol and phospholipids onto apoA1 led to a proposal that ABCA1 mediates the transbilayer externalization of phospholipids, an activity integral not only to the formation of HDL particles but also to another, distinct process: the recognition and clearance of apoptotic cells by macrophages. Expression of phosphatidylserine (PS) on the surface of both macrophages and their apoptotic targets is required for efficient engulfment of the apoptotic cells, and it has been proposed that ABCA1 is required for transbilayer externalization of PS to the surface of both cell types. To determine whether ABCA1 is responsible for any of the catalytic activities known to control transbilayer phospholipid movements, these activities were measured in cells from *ABCA1^−/−^* mice and from Tangier individuals as well as ABCA1-expressing HeLa cells. Phospholipid movements in either normal or apoptotic lymphocytes or in macrophages were not inhibited when cells from knockout and wildtype mice or immortalized cells from Tangier individuals vs normal individuals were compared. Exposure of PS on the surface of normal thymocytes, apoptotic thymocytes and elicited peritoneal macrophages from wildtype and knockout mice or B lymphocytes from normal and Tangier individuals, as measured by annexin V binding, was also unchanged. No evidence was found of ABCA1-stimulated active PS export, and spontaneous PS movement to the outer leaflet in the presence or absence of apoA1 was unaffected by the presence or absence of ABCA1. Normal or Tangier B lymphocytes and macrophages were also identical in their ability to serve as targets or phagocytes, respectively, in apoptotic cell clearance assays. No evidence was found to support the suggestion that ABCA1 is involved in transport to the macrophage cell surface of annexins I and II, known to enhance phagocytosis of apoptotic cells. These results show that mutations in ABCA1 do not measurably reduce the rate of transbilayer movements of phospholipids in either the engulfing macrophage or the apoptotic target, thus discounting catalysis of transbilayer movements of phospholipids as the mechanism by which ABCA1 facilitates loading of phospholipids and cholesterol onto apoA1.

## Introduction

Mutations in the ATP-binding cassette (ABC) ATPase, ABCA1, are responsible for Tangier disease [1]–[3], an autosomal recessive disorder characterized by the virtual absence of high-density lipoprotein (HDL), reduced levels of low-density lipoprotein (LDL), and mildly elevated triglycerides. Mice in which the ABCA1 gene has been disrupted (*ABCA1^−/−^*) are unable to load cholesterol and phospholipids to apoA-1, implicating ABCA1 in the efflux of these lipids from cells [4]–[6]. Remarkably, mutations in ABCA1 have been reported to give rise to a seemingly unrelated phenotype, a defect in the clearance of apoptotic cells by macrophages [7]. When cells undergo apoptosis or programmed cell death, they must distinguish themselves from their healthy counterparts in order to be safely eliminated. A number of molecules on the apoptotic cell surface have been implicated as ligands for a wide variety of receptors on the macrophage surface [8], [9]. However, ABCA1, a membrane-spanning enzyme that is not an obvious candidate as either ligand or receptor, has been identified as necessary for efficient engulfment of apoptotic cells. When antibodies to this ATPase are introduced into macrophages, their ability to engulf apoptotic thymocytes is severely compromised [10].

The evidence for involvement of an ABC ATPase in the recognition of mammalian apoptotic cells echoes findings from genetic studies of cell death in the nematode, *C. elegans*. Engulfment in *C. elegans* is controlled by a group of *ced*, or cell death genes [11]. One of these genes, *ced-7*, encodes an ABC protein [12]. Interestingly, efficient clearance of corpses requires expression of *ced-7* in both apoptotic germ cells and the gonadal sheath cells that engulf them [12].

This curious requirement for the same factor at the surface of both target and phagocyte is mirrored by another molecule. Redistribution of phospholipids across the plasma membrane of both apoptotic targets and phagocytes plays a crucial role in engulfment. Phospholipids are normally distributed asymmetrically across the membrane bilayer, with the aminophospholipids, phosphatidylserine (PS) and phosphatidylethanolamine (PE), concentrated in the inner leaflet, and the neutral phospholipids, phosphatidylcholine (PC) and sphingomyelin, concentrated in the outer leaflet. This distribution is maintained by an aminophospholipid translocase, a P-type ATPase [13] that actively and specifically transports the aminophospholipids from the outer to the inner leaflet of the bilayer [14]. When lymphocytes undergo apoptosis, they inactivate this enzyme and activate a non-specific lipid flipsite, termed the scramblase, which promotes rapid equilibration of phospholipids across the bilayer, producing a symmetric distribution [15]. When PS, which is normally restricted to the inner leaflet of the plasma membrane, reaches the surface of the cell, it serves as a recognition signal for macrophage engulfment [8], [16]. On the other hand, macrophages constitutively express low levels of PS on their surface [17], which is acquired as monocytes differentiate into macrophages [18]. Interestingly, ABCA1 expression is upregulated during monocyte/macrophage differentiation [19]. PS on both the apoptotic lymphocyte and the macrophage is required for phagocytosis of the apoptotic cell, since phagocytosis is inhibited by masking the PS on either of the partner cells using the small protein, annexin V, whose Ca^2+^-dependent binding is PS-specific [17], [18], [20].

The dual requirement for *ced-7* and the dual requirement for cell surface PS for apoptotic cell engulfment raise the possibility that the two are related; in mammals, this hypothesis predicts that ABCA1 plays a role in the exposure of PS on both the apoptotic lymphocyte and the macrophage. Tests of this hypothesis have been performed. The scramblase, responsible for exposure of PS on apoptotic lymphocytes, can be artificially activated in the absence of apoptosis by elevating cytosolic levels of Ca^2+^ using a Ca^2+^ ionophore [21], [22]. PS exposure by this process in both lymphocytes and macrophages is inhibited by glyburide or oligomycin, chemical inhibitors of ABC transporters [23]. Furthermore, when either apoptotic lymphocytes or macrophages are treated with the inhibitors, phagocytosis is inhibited [23]. However, these experiments cannot prove that ABCA1 is responsible for PS exposure. The inhibitors are not specific to ABCA1 and their effects might result directly or indirectly by inhibiting something else, including some other ABC protein.

A more specific test for ABCA1 function is provided by cells from *ABCA1^−/−^* mice and from Tangier patients homozygous for mutations that disrupt expression of the human ABCA1 gene. One study using *ABCA1^−/−^* mice reported a reduction in PS exposure on the surface of red blood cells in which the scramblase had been activated by Ca^2+^ and on apoptotic lymphocytes as assessed by annexin V binding [7]. Here, we examine the role of ABCA1 in phospholipid externalization in detail, measuring a wide variety of transbilayer movements in cells from *ABCA1^−/−^* mice and from Tangier individuals as well as ABCA1-expressing HeLa cells, including translocation of phospholipids across the plasma membrane by the aminophospholipid translocase, both spontaneous (basal) and scramblase-mediated movement of PS from the inner to the outer leaflet of the plasma membrane, and the steady-state exposure of PS on the cell surface. In addition, we test the ability of ABCA1-deficient macrophages to phagocytose apoptotic cells and the ability of ABCA1-deficient apoptotic lymphocytes to serve as targets for macrophages. Finally, we test the hypothesis that ABCA1 is required for exposure of annexins on the macrophage surface, proteins previously shown to be involved in phagocytosis of apoptotic lymphocytes [24].

## Results

**Figure 1:**
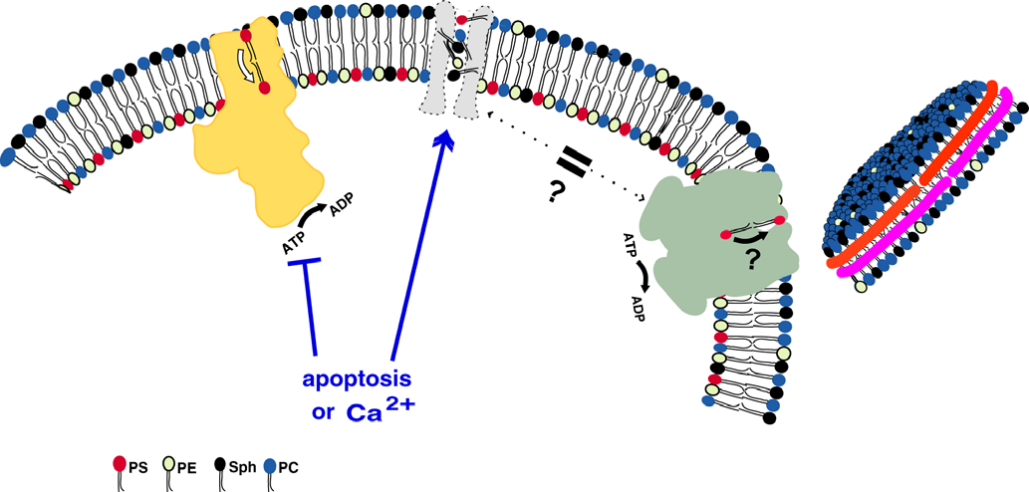
Known and potential transbilayer lipid transporters. The aminophospholipid translocase (left) and ABCA1 (right) proteins are drawn approximately to scale in outline forms taken from atomic structures of a P-type ATPase (the Ca^2+^ transporter;[54]) and an ABC protein (BtuCD,[55]). The molecular shape of the scramblase (center) is arbitrary, since the protein responsible for this activity is not known. One possibility, investigated here, is that the ABCA1 protein is actually the protein responsible for this activity; another possibility is that the ABCA1 protein transports PS from the inner to the outer leaflet, as depicted. An HDL particle (far right) is also drawn approximately to scale by extrapolation from the structure of other lipoprotein particles, with the apoA1 protein drawn as the belt-like helices enclosing the lipid core. The molecular shape of an unlipidated apoA1 protein is unknown.

The distribution of phospholipids across the plasma membrane is regulated by at least two opposing activities. One is active transport of PS, and to a lesser extent PE, from the outer to the inner leaflet by the aminophospholipid translocase; the other is equilibration of all phospholipids between the inner and outer leaflets by the scramblase. ABCA1 could affect phospholipid externalization if it were one of these two enzymes, if it regulated one of these activities, or if it supplemented these activities. These possibilities are outlined in Figure 1, and systematically investigated below.

### Translocase Activity

**Figure 2:**
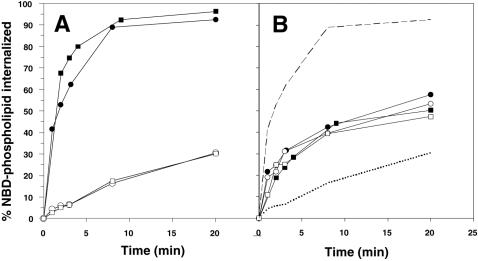
Internalization (translocation) of NBD-labeled phospholipids by normal human fibroblasts and fibroblasts from Tangier individuals. The outer leaflet of the plasma membrane of normal (circles) or Tangier (squares) fibroblasts was labeled with NBD-PC (open symbols) or NBD-PS (filled symbols). At various times samples were removed into BSA to extract outer leaflet probe and remaining inner leaflet probe was measured by flow cytometry and expressed as percent transported. Measurements were made at room temperature. (A) Viable fibroblasts, (B) spontaneously apoptotic fibroblasts. Dashed and dotted lines in B have been redrawn from A for transport by normal fibroblasts.

In healthy normal cells, the aminophospholipid translocase activity rapidly transports PS from the outer to the inner leaflet of the plasma membrane bilayer, in contrast to the slower, unaided diffusion of PC across the same bilayer. In apoptotic cells this activity is down-regulated. ABCA1 might contribute to PS exposure on the cell surface if it reduced or blocked translocase activity. If so, ABCA1 mutations would be expected enhance translocase activity in healthy cells, or to no longer allow the translocase to be inactivated in apoptotic cells. To measure translocase activity, fluorescent NBD-PS or NBD-PC derivatives are introduced into the outer leaflet of cells, and the kinetics of probe transfer to the inner leaflet measured periodically by first removing probe still on the surface with BSA-containing buffers and then quantitating the remaining, internalized probe by measuring cellular fluorescence using flow cytometry. Figure 2A shows the results of applying this assay to primary fibroblasts from normal and Tangier individuals. Internalization of PC was slow for both normal and mutant nonapoptotic cells. This rate provides an upper boundary for probe internalization by endocytosis, although measurements of internalization of other probes such as sphingomyelin (not shown) indicate that PC internalization overestimates the contribution of endocytosis. In contrast, internalization of PS was rapid in both normal and Tangier cells, indicating that disabling the ABCA1 gene does not enhance PS internalization by the aminophopholipid translocase in these cells. Similar results were observed in comparisons of translocase activity in normal vs Tangier EBV-transformed B lymphocytes, in thymocytes from wildtype vs *ABCA1^−/−^* mice, and in control human HeLa cells, which do not express detectable levels of ABCA1 protein, or transfected HeLa cells expressing functional ABCA1 protein [25](data not shown).. These results show that ABCA1 is not the aminophospholipid translocase, and suggest that it is also not a negative regulator of the translocase, since its absence does not enhance and its overexpression does not reduce translocase activity (see Table 1 for a summary of these and following results). These results leave open the possibility that ABCA1 is required to downregulate the translocase in apoptotic cells. In apoptotic cells, not only translocase activity but also transbilayer lipid movements catalyzed by the scramblase must be considered in PS externalization. Therefore, whether ABCA1 is or regulates the scramblase was addressed.

**Table 1:** Summary of Assays

*Cell type*	Activity Measured				
	*Translocase*	*Scramblase*		*PS-specific externalization*	*Phagocytosis*
	*NBD-PL internalization*	*Continuous AxV binding*	Static AxV binding	*Externalization of internalized NBD-PS*	
***Tangier vs. normal***					
*B-lymphocytes*	no increase	no decrease (Ca^2+^-activated )	no decrease (apoptosis-activated)	no decrease (basal)	no decrease (T)
*Fibroblasts*	no increase	no decrease (Ca^2+^-activated )	ND	no decrease (basal µ ± ApoA1)	ND
***ABCA1^−/−^ vs. wt***					
*Thymocytes*	no increase	no decrease (Ca^2+^-activated )	no decrease (apoptosis activated)	no decrease (basal or Ca^2+^-activated)	no decrease (T)
*Macrophages*	no increase	no decrease (Ca^2+^-activated )	no decrease (constitutive)	no decrease (basal)	no decrease (P)
***HeLa expressing ABCA1 vs. control***	no increase	no increase (Ca^2+^-activated )	no increase (Ca^2+^-dependent)	no increase (basal)	ND

ND = Not Determined T = as targets P = as phagocytes

### Ca^2+^-activated Scramblase Activity

Normally, the basal level of movement of PS from the inner to the outer leaflet of the plasma membrane, is slow; activation of the scramblase enhances the rate of PS externalization. When Ca^2+^ and a Ca^2^-ionophore are added to normal human or mouse lymphocytes at levels that result in elevation of cytosolic Ca^2+^ concentrations within a few seconds, scramblase-mediated lipid movement begins after a lag of a minute or more [22]. Net PS exposure can be measured continuously using fluorescently-labeled annexin V; in the presence of this probe, PS appearance on the cell surface results in binding of annexin V and an increase in cellular fluorescence which can be measured in the flow cytometer continuously as a function of time. This assay was used to demonstrate that extensive exposure of thymocytes to glyburide, an inhibitor of ABC proteins, prevents exposure of PS on the cell surface when cytosolic Ca^2+^ concentrations are elevated [23]. Glyburide could inhibit PS exposure by blocking some step in the lag phase, and thereby prolonging the onset of lipid movement indefinitely, or by blocking lipid movement itself, after completion of steps in the lag phase. To distinguish these possibilities, 50 µM glyburide was added to Jurkat T lymphocytes growing in suspension culture; at intervals, aliquots were removed and the ability of the cells to activate the scramblase was tested by measuring PS exposure after addition of Ca^2+^ and ionophore. At a concentration of 50 µM, glyburide inhibition of scramblase activity was complete by 30 min after addition of the inhibitor to cells (data not shown). At earlier times during this incubation, inhibition was observable, but incomplete. When the kinetics of PS exposure were examined at these intermediate times, it was apparent that the lag time before the onset of lipid movement remained relatively constant, but that once the lag was over, the rate at which PS moves to the external leaflet was slowed (data not shown). These results show that the effect of glyburide is not on the preliminary step(s) that occur during the lag phase, but rather on lipid movement itself, consistent with a role for an ABC protein in the process of PS externalization. However, there are many potential targets for glyburide which might account for its effects on this process. A more definitive test of whether ABCA1 is required for PS externalization is whether there is a detectable inhibition of scramblase-mediated phospholipid movements in ABCA1-deficient cells.

**Figure 3:**
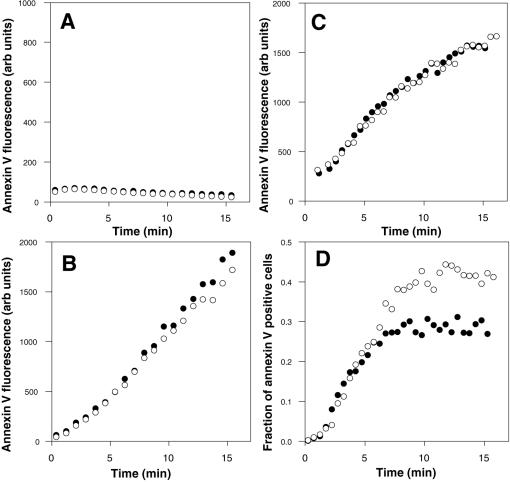
Endogenous PS externalization in normal and ABCA1-deficient cells measured by continuous annexin V binding assay. (A) Normal or Tangier human fibroblasts, (B) EBV-transformed normal or Tangier B lymphocytes, or (C and D) thymocytes from wildtype or *ABCA1^−/^* ^−^ mice were treated with Ca^2+^ and Ca^2+^ ionophore, and cellular fluorescence in the presence of fluorescent annexin V measured continuously over time at room temperature. Normal/wildtype, filled circles; ABCA1-deficient, open circles. A–C, median fluorescence of cells; D, fraction of cells stained by fluorescent annexin.

It has been reported that Ca^2+^-induced externalization of PS is inhibited in primary fibroblasts isolated from *ABCA1^−/−^* mice, as measured by a reduced fraction of cells binding annexin V over time compared to wildtype controls [7]. These data suggest that ABCA1 is required for activation of scramblase activity. Indeed, as shown in Figure 3A, no Ca^2+^-activated PS externalization could be detected in primary fibroblasts from Tangier individuals. However, normal fibroblasts with a functional ABCA1 activity (as indicated by lipid transfer to apoA1, not shown) also do not expose PS in response to Ca^2+^ (Figure 3A). This absence of Ca^2+^ activation of scramblase activity was observed in a variety of fibroblast and epithelial cell lines (CHO, BHK, 3T3, MCF-7, HeLa) and other non-hematopoietic cell types (data not shown), suggesting that the ability to activate the scramblase with Ca^2+^ is most developed in red blood cells, platelets, lymphocytes, and macrophages. Since Ca^2+^ does not activate scramblase activity in HeLa cells and HeLa cells do not express detectable levels of ABCA1 protein [25], it was asked whether expressing ABCA1 in HeLa cells could produce Ca^2+^-activated scrambling. However, no activity was seen (data not shown), indicating that elevation of ABCA1 expression does not repair the absence of Ca^2+_^activated PS externalization in either HeLa cells or fibroblasts.

The fact that ABCA1 alone is not sufficient to confer Ca^2+^-dependent scramblase activity does not rule out the possibility, suggested by the studies with glyburide ([7] and see above), that it is nevertheless required in those cells in which such activation occurs. As shown in Figure 3B, Ca^2+^-activated PS externalization does occur in EBV-transformed human B lymphocytes (Figure 3B); however, it also occurs at identical rates in EBV-transformed human B lymphocytes from Tangier individuals. To ensure that these results were not peculiar to the human system or to cultured cell lines, similar experiments were carried out with primary mouse cells. As shown in Figure 3C, PS externalization by Ca^2+^-activated scramblase activity proceeded at identical rates in primary thymocytes from wildtype and *ABCA1^−/−^* mice, indicating that human and murine lymphoid cells behave identically in this respect. In the original report suggesting that Ca^2+^ induced exposure of PS was sensitive to ABCA1 deletion [7], although a similar assay was used, the result was not reported as the level of PS exposure, as shown here, but rather the fraction of cells in which PS exposure was elevated. The latter measure is more sensitive to changes in the efficiency of scramblase activation, and less sensitive to changes in the rate of lipid movement. To ensure that the difference in measures reported (fraction of PS-exposing cells vs level of PS exposure) was not responsible for the difference in results compared to the previous study, the fraction of PS-exposing cells was determined. As shown in Figure 3D, the rate at which cells become activated is not slower in cells from the *ABCA1^−/−^* mice. In addition, the fraction of responsive cells is not reduced; indeed, in the experiment shown this fraction was slightly elevated in cells from the knockout mice, although in repeated experiments, this result was not generally observed. Together, these results demonstrate that deletion of ABCA1 does not reduce the efficiency of scramblase activation, and does not reduce the rate of PS movement catalyzed by the scramblase following elevation of cytosolic Ca^2+^ levels in either human or mouse cells.

### Apoptosis-induced Scramblase Activity

**Figure 4:**
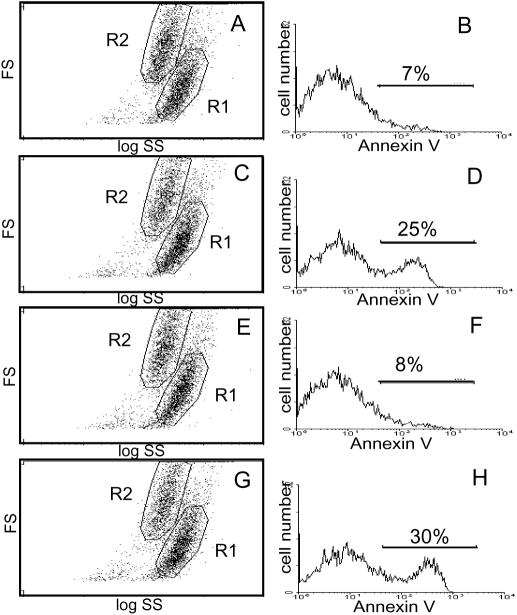
Endogenous PS externalization in apoptotic EBV-transformed human B lymphocytes. EBV-transformed normal (A–D) or Tangier (E–H) B lymphocytes were untreated (A&B, E&F) or treated with camptothecin (C&D, G&H) to induce apoptosis, stained with fluorescent annexin V, and examined by flow cytometry. Left panel, forward (FS) vs side (SS) light scatter plots, with cells of normal size and shape in the R2 gate and shrunken cells in the R1 gate. Right panel, fluorescence profile of cells in the R2 gate.

Studies with cells from patients with Scott disease, in which Ca^2+^ activation of the scramblase has been inactivated by mutation, suggest that the scramblase activated during apoptosis may be the same as the scramblase activated by elevated cytoplasmic Ca^2+^, but that the activation mechanisms are distinct [22]. If so, it is possible that ABCA1 is only required for PS exposure during apoptosis, which would explain the inability of ABCA1 deletion to block Ca^2+^-induced lipid movement in normal, healthy cells. The activation of scramblase during apoptosis is most easily seen in the change in NBD-PS and NBD-PC movement observed in apoptotic cells. Apoptotic cells can be identified by their characteristic light scattering properties (see Figure 4, below), so that internalization of phospholipid by just those cells can be measured using flow cytometry. In contrast to healthy cells where the aminophospholipid translocase transports NBD-PS (but not NBD-PC) rapidly to the inner leaflet (see Figure 2A), in apoptotic fibroblasts, both phospholipids moved to the cell interior at the same intermediate rate (Fig 2B). The same change in pattern, and the same resulting rates of lipid movement, were observed whether the fibroblasts were normal or Tangier apoptotic fibroblasts, indicating that ABCA1 deletion does not inhibit this process. Similar results were seen with EBV-transformed apoptotic normal and Tangier B lymphocytes, as well as with apoptotic normal and ABCA1-expressing HeLa cells (data not shown), indicating that ABCA1 is not required for activation of the scramblase in apoptotic cells, nor does it contribute positively or negatively to the rate at which phospholipids move across the bilayer in apoptotic lymphocytes or fibroblasts. In addition, the failure to observe accumulation of PS in the interior of ABCA1-deficient apoptotic cells shows that the translocase has been inactivated as usual, showing that ABCA1 is not required for down-regulation of the translocase activity.

Although these data indicate that the scramblase is activated during apoptosis in cells lacking ABCA1, it is possible that ABCA1 is still necessary for this activation to be efficient. Cells in a population do not enter apoptosis synchronously; induction of lipid movements is therefore not synchronous, as it is for Ca^2+^-activated induction. As a result, the efficiency of scramblase induction during apoptosis cannot be measured by the continuous annexin V binding assays described above. However, it is possible to measure the rate of activation of PS exposure relative to other events in the apoptotic program, using conventional annexin V binding assays. Apoptotic cells undergo measurable cell shrinkage. If PS exposure is induced before this change in size, annexin V binding is observed in cells of normal size and shape, as well as in the smaller and more irregularly shaped apoptotic cells produced by shrinkage. This pattern can be readily detected in the flow cytometer, as shown for EBV-transformed apoptotic B lymphocytes in the left panels of Figure 4(A,C,E,G). Cells in the gated R2 subpopulation are of normal size and shape, while cells which have undergone shrinkage appear in the gated R1 subpopulation of lower forward angle light scatter and slightly higher side scatter. At early times after the induction of apoptosis, PS-exposing cells, which are usually absent from the normal (R2) subpopulation (Fig 4B), begin to appear (Fig 4D). If inactivation of ABCA1 substantially slowed the onset of lipid rearrangements, more cells would be expected to complete the shrinkage step before the activation of lipid movement. The result would be a substantial drop in the number of PS-exposing cells in the subpopulation of cells with normal morphology. As shown in Figure 4 (D compared to H), this expectation is not observed; in fact in this example, normal-sized cells which expose PS and bind annexin V appear at a slightly higher frequency in the absence of ABCA1. These results suggest that deletion of ABCA1 does not reduce the efficiency of scramblase activation in apoptotic cells.

### PS-specific Outward Lipid Movement

**Figure 5:**
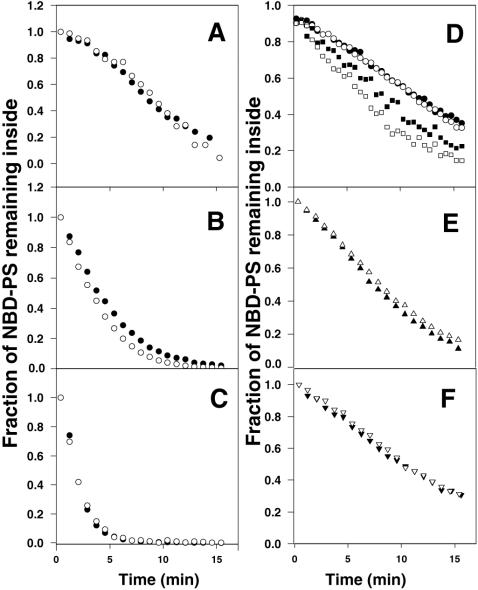
Basal (or Ca^2+^-activated) NBD-PS externalization in normal and ABCA1-deficient cells. (A) Normal human or Tangier fibroblasts, (B) EBV-transformed normal or Tangier B lymphocytes, (C) Control or ABCA1-GFP-transfected HeLa cells, (D) thymocytes from wildtype or *ABCA1^−/−^* mice, (E) normal human fibroblasts or (F) Tangier fibroblasts were allowed to internalize NBD-PS, dithionite added to reduce and render non-fluorescent externalized NBD-PS, and cellular fluorescence (unexternalized NBD-PS) measured continuously over time at room temperature. (A–D) normal/wildtype, filled symbols; ABCA1-deficient or replete (HeLa), open symbols. (D) untreated, circles; treated with Ca^2+^ and Ca^2+^ ionophore, squares. (E and F) presence (open triangles) or absence (closed triangles) of apoA1.

It has been suggested that ABCA1 contributes to PS exposure, not through the scramblase, but rather by specific enhancement of movement of just PS from the inner to the outer leaflet [26](see Discussion). As shown in Figure 3, the presence or absence of ABCA1 makes no detectable difference in the rate at which PS externalization occurs in cells in which the scramblase is activated by Ca^2+^. It could be that the rate of externalization of PS induced by Ca^2+^ is too fast to see the effects of additional, ABCA1-dependent, PS movement. One might, therefore, measure PS externalization in cells in which the scramblase was not activated, except that, as shown in Figure 2, ABCA1-dependent PS movement to the external leaflet would be masked by the action of the translocase. However, a method has been devised to measure PS externalization rates irrespective of an active translocase. In this assay, healthy cells were allowed to internalize NBD-PS by means of the translocase, and were then placed in the presence of the reducing agent sodium dithionite. In the presence of this reagent, internalized NBD-PS which reappears at the cell surface is immediately reduced to a non-fluorescent amino derivative. The resulting decrease in cell fluorescence with time thus measures the rate at which the PS analog moves from the inner leaflet to the cell surface. As shown in Figure 5, NBD-PS moved from the cell interior to the cell surface in lymphocytes, fibroblasts, or HeLa cells. These observed basal rates differ significantly from one cell type to another, ranging from a half time for externalization of about 8 min in B lymphocytes to about 2 min in HeLa cells; importantly, these times are all slow by comparison with the half time for probe reduction by dithionite (about 5 sec). These rates are not increased by increasing the concentration of added dithionite (data not shown), indicating that they are not the result of membrane permeability to dithionite. There are no differences in the externalization rates between normal and Tangier fibroblasts (Figure 5A) or B lymphocytes (Fig 5B), or between normal and ABCA1-expressing HeLa cells (Fig 5C), showing that the basal PS externalization process in all of these cells is independent of ABCA1. Because ABCA1 has been reported to directly bind apoA1 [27], the possibility was considered that ABCA1 catalyzed lipid exchange only in response to the presence of apoA1. As shown in Figure 5E and 5F, however, the presence of apoA1 had no effect on the rate of PS externalization, suggesting that apoA1 binding to ABCA1 does not result in active PS externalization.

Because this assay measures phospholipid externalization directly, versus indirectly by annexin V binding, it might be considered a more valid assay for detecting active PS-specific externalization. Although the assay cannot be applied to apoptotic cells, since they are permeable to dithionite (unpublished observation), it can be used to measure Ca^2+^-induced PS externalization. As shown in Figure 5D, the assay reveals that a substantial rate of PS externalization takes place in primary mouse thymocytes without scramblase activation; as in the case of human lymphocytes and fibroblasts (Fig 5 A–C), the rate was unaltered in ABCA1-deficient cells. Upon activation of scramblase activity by Ca^2+^, this rate increased as expected (Figure 5D). Notably, however, the increase in the rate of PS externalization was seen in both wild type and ABCA1-deficient cells, and the increase was even greater in the mutant compared to normal cells. These results indicate that ABCA1 does not contribute to any measurable avenue of Ca^2+^-activated PS externalization in these cells.

### ABCA1 Function in Macrophages

**Figure 6:**
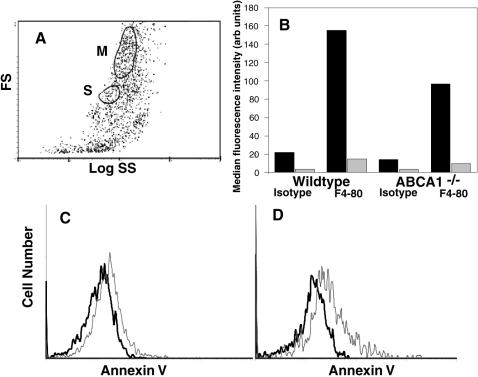
Identification and annexin V staining of macrophages in peritoneal lavage from wildtype or *ABCA1^−/−^* mice. Cells from peritoneal lavage were stained with mAb F4/80 (or isotype control mAb) or with annexin V on ice and examined by flow cytometry at room temperature. A, forward (FS) vs side (SS) light scatter plot, with cells having typical macrophage light scatter characteristics in the M gate and Asmall@ cells in the S gate. B, median fluorescence intensity of cells in the M gate (black) or S gate (grey) following staining with mAb F4/80 or isotype control mAb. C&D, annexin V staining, in the presence (thin line) or absence (thick line) of Ca^2+^, of cells in the M gate from wildtype (C) or *ABCA1^−/−^* (D) mice.

ABCA1 is present and active in macrophages [28], consistent with a role for the protein in both cholesterol export and apoptotic cell removal, since both of these functions are important in macrophages. It is thus possible that the role of ABCA1 in lipid movements is specifically observable in macrophages. To investigate this possibility, elicited peritoneal macrophages from wildtype and *ABCA1^−/−^* mice were examined. Although the exudate cell population is quite heterogeneous, viable macrophages can be identified with mAb antibody F4/80[29]; F4/80-positive cells occupy a discrete region, gated subpopulation M, in the forward light scatter/side light scatter profile of the entire cell population (Figure 6A and 6B). F4/80-negative cells are also present, such as gated subpopulation S in Figure 6A and 6B. Cells in both the M and S regions exclude propidium iodide, indicating that they retain an intact plasma membrane.

The cells in the S subpopulation resemble most normal healthy cells in that they fail to bind annexin V (data not shown), implying that the lipids in the plasma membrane are asymmetric, with PS confined to the inner leaflet. It has been reported that expression of ABCA1 in HeLa cells results in a small, but measurable increase in binding of annexin V to the surface of normal healthy cells, suggesting that ABCA1 expression may result in low level PS exposure in these cells [7]. This result is reminiscent of the observation that the lipid distribution in the plasma membrane of macrophages differs from that of most other normal healthy cells in that PS is constitutively expressed at low levels on the surface of viable, non-apoptotic macrophages, where it is required for efficient recognition of PS-exposing apoptotic target cells [17]. We therefore asked whether this macrophage-specific PS exposure was ABCA1-dependent. As shown in Figure 6C, macrophages in the M region display this low level of Ca^2+^-dependent annexin V binding, consistent with the presence of low levels of PS exposed on their surface. However, as shown in Figure 6D, Ca^2+^-dependent binding of annexin V is not reduced in macrophages from *ABCA1^−/−^* mice, indicating that this characteristic is not ABCA1-dependent. In view of these results, annexin V binding by healthy HeLa cells expressing ABCA1 was examined and the data confirmed that these cells do bind slightly more annexin V than control HeLa cells. However, this binding was not Ca^2+^-dependent (data not shown). Since Ca^2+^ dependence is the hallmark of PS-specific annexin binding, this result does not reflect increased PS exposure, and is therefore unrelated to constitutive PS exposure in macrophages.

**Figure 7 pone-0000729-g007:**
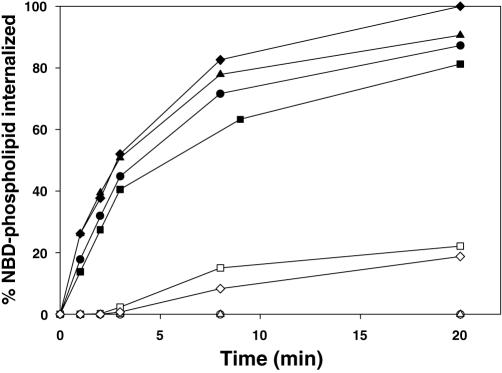
Internalization (translocation) of NBD-labeled phospholipids by peritoneal macrophages from wildtype and *ABCA1^−/−^* mice. The outer leaflet of the plasma membrane of macrophages from wildtype (diamonds) or *ABCA1^−/−^* (squares) mice was labeled with NBD-PC (open symbols) or NBD-PS (closed symbols). At various times samples were removed into dithionate to reduce outer leaflet probe and after 5 min remaining inner leaflet probe was measured by flow cytometry at room temperature and expressed as percent transported. Cells from wildtype (triangles) and *ABCA1^−/−^* (circles) mice in the S gate in Figure 6 are shown for comparison.

Annexin V binding by macrophages reveals the steady-state presence of PS in the outer leaflet of the plasma membrane, but provides no information on the transbilayer lipid movements that underlie this distribution. Measuring these movements with NBD-labeled phospholipids as was done above with other cell types can be problematic with macrophages, which rapidly internalize membrane by endocytosis. This rapid bulk membrane flow nonspecifically internalizes any lipid probe present in the membrane, sequestering even externally-directed probes in the lumenal leaflet of a vesicle. This process does not require, and can even mask, transbilayer lipid transport. Sodium dithionite was used to overcome this problem. After addition of NBD-labeled lipids and incubation to allow probe internalization as usual, samples of labeled macrophages taken at various times were incubated for 5 min with dithionite at room temperature to allow internalization of the reducing agent into the lumen of endocytic vesicles. During this secondary incubation, any probe remaining in the medium or outer leaflet will be reduced to the nonfluorescent form. In addition, reducing agent in the lumen of endocytic vesicles will enter the endocytic pathway and when these vesicles fuse with early endosomes, dithionite will come into contact with previously endocytosed probe which is still restricted to the (lumenal) leaflet. This process could reduce fluorescence from probe molecules that have been sequestered by endocytosis. To test whether this approach makes it possible to measure headgroup-specific transbilayer transport of phospholipids, the protocol was used to compare the internalization of PC and PS in macrophages. As shown in Figure 7, the assay revealed the standard aminophospholipid translocase pattern of rapid PS internalization and much slower PC internalization, comparable to that seen in the control S cells in the same experiment. As with other cell types, the macrophage translocase activity is unaffected by disabling ABCA1. In this case, the rate of internalization of PC by macrophages is measurable compared to no measurable transport in the control S subpopulation. Uptake of PC by macrophages includes both probe taken up by transport across the bilayer, and probe taken up by endocytosis but not reduced by internalized dithionite. This rate therefore defines an upper limit for the background of nonspecific endocytic uptake of probe still in the external leaflet but not reduced during the secondary dithionite incubation.

**Figure 8 pone-0000729-g008:**
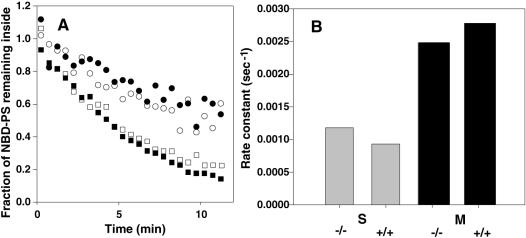
Basal NBD-PS externalization in peritoneal macrophages from wildtype or *ABCA1^−/−^* mice. Macrophages (squares) or cells in the S gate in Figure 6 (circles) from wildtype (filled symbols) or *ABCA1^−/−^* (open symbols) mice were allowed to internalize NBD-PS, dithionite was added to reduce and render non-fluorescent externalized NBD-PS, and cellular fluorescence (unexternalized NBD-PS) was measured continuously over time (A). Rate constants derived by simple exponential fit to the data for macrophages (black) and gated S cells (grey) are compared in (B).

By using this protocol to load cells with NBD-PS, the rate of PS externalization by macrophages from wildtype and *ABCA1^−/−^* mice could be measured. As shown in Figure 8, the basal rate of PS externalization is faster in macrophages (M region cells) than in non-macrophages (S region cells) measured in the same population. The rate for macrophages is comparable to that measured for lymphocytes, for example (see Fig 5, above). More importantly, the rate is similar whether macrophages are from wildtype or *ABCA1^−/−^* mice, indicating that this form of outward transbilayer PS movement is independent of ABCA1.

**Figure 9:**
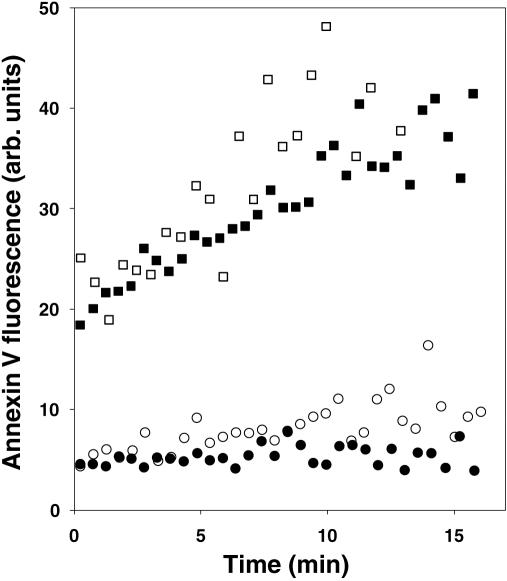
Ca^2+^-activated endogenous PS externalization in peritoneal macrophages from wildtype or *ABCA1^−/−^* mice measured by continuous annexinV binding assay. Macrophages (squares) or cells in the S gate in Figure 6 (circles) from wildtype (filled symbols) or *ABCA1^−/−^* (open symbols) mice were treated with Ca^2+^ and Ca^2+^ ionophore, and cellular fluorescence measured continuously over time at room temperature.

Whether the ablation of ABCA1 disables scramblase-mediated lipid movement in macrophages could be tested by either activating the scramblase with Ca^2+^ or by using apoptotic macrophages. Due to the complexity of the light scatter distribution of the peritoneal lavage preparation, the apoptotic macrophage population could not be uniquely identified for separate analysis. Therefore, the continuous annexin V binding assay described above was used to measure Ca^2+^-induced externalization of PS by macrophages. As shown in Figure 9, elevation of cytosolic Ca^2+^ induced the immediate appearance of additional PS on the macrophage surface; in contrast, scramblase in the non-macrophage cells in the S region could not be activated by Ca^2+^, much as was observed in fibroblasts (Fig 3A). Importantly, as was shown with the lymphocytes above, the scramblase in macrophages also showed no defect, either in activation or rate of PS appearance, when cells from *ABCA1^−/−^* mice were compared with wildtype. These results indicate that ABCA1 is not a macrophage-specific form of the scramblase. Combined with the absence of an ABCA1 requirement for uninduced basal PS externalization, these results show that ABCA1 does not contribute to any measurable mechanism for PS externalization in macrophages.

**Figure 10:**
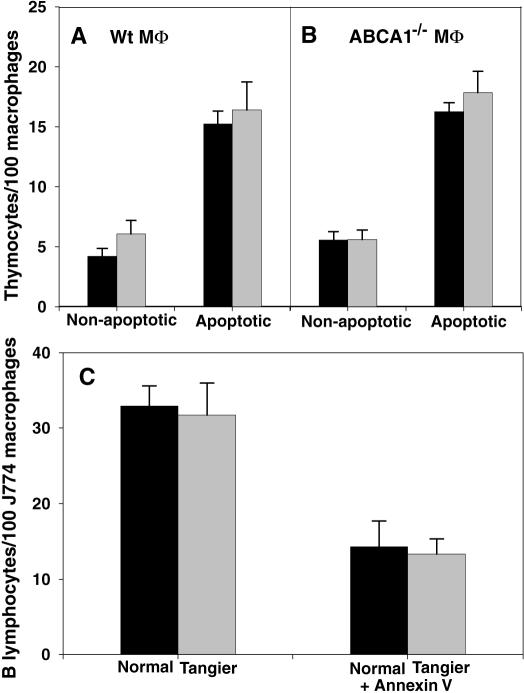
Phagocytosis of apoptotic targets by wildtype or *ABCA1^−/−^* macrophages. A&B, phagocytosis of non-apoptotic or apoptotic thymocytes from wildtype (black) or *ABCA1^−/−^* (gray) mice by peritoneal macrophages from wildtype (A) or *ABCA1^−/−^* (B) mice. C, phagocytosis of camptothecin-induced apoptotic EBV-transformed normal (black) or Tangier (gray) human B lymphocytes, either untreated or pre-treated with 10 µM Annexin V, then washed prior to presentation to mouse J774 macrophages. Error bars represent the standard error of 3 replicates.

Given the absence of detectable reductions in transbilayer lipid movements correlated with the deletion of ABCA1 in either elicited peritoneal macrophages or their potential apoptotic targets (see Table 1 for complete summary), whether a defect in apoptotic cell recognition could be detected when ABCA1 was disabled in either the recognizing macrophage, the apoptotic target, or both, was examined. For this purpose, a standard phagocytosis assay was used in which an excess of target apoptotic thymocytes were presented to elicited macrophages immobilized on glass coverslips. The number of engulfed target cells was then measured at 30 min when the rate of target cell uptake is linear, so that the assay measures the rate of recognition and engulfment [30]. As shown in Figure 10A and B, dexamethasone induction of apoptosis in the target cells results in a readily measurable increase in the rate at which wildtype thymocytes are engulfed by wildtype macrophages; freshly isolated thymocytes spontaneously develop some apoptotic cells, explaining the presence of some uptake using uninduced cells as targets. The rate of engulfment is not reduced when either the target cells, the macrophages, or both are derived from *ABCA1^−/−^* mice, indicating that this rate is not sensitive to the presence of ABCA1 in this system. A comparable experiment was not possible for the human system because Tangier and normal macrophages were not readily available. However, EBV-transformed normal and Tangier B lymphocytes were used as targets for wild-type macrophages of the J774 mouse cell line. As shown in Figure 10C, phagocytosis of apoptotic cells was not sensitive to the ABCA1 gene in the apoptotic target cells. Recognition and engulfment of apoptotic cells in this system is dependent on PS expression, as shown by the sensitivity of the engulfment to masking PS with annexin V (Figure 10C).

**Figure 11:**
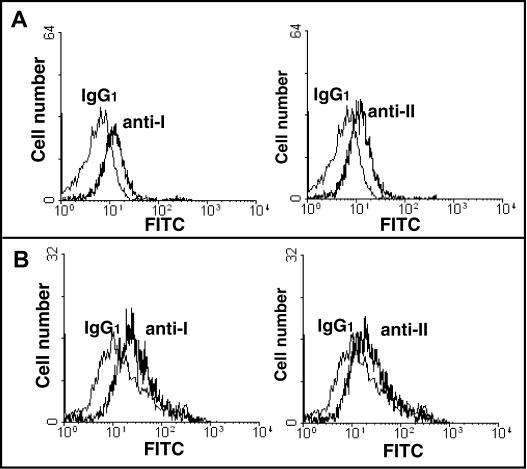
Binding of anti-annexin mAbs to peritoneal macrophages from wildtype or *ABCA1^−/−^* mice. Macrophages from wildtype (A) or *ABCA1^−/−^* (B) mice were stained with anti-annexin I, II or mouse IgG1 isotype control mAbs and analyzed by flow cytometry.

Recognition and engulfment of target apoptotic cells is facilitated by the presence of annexins I and II, on the surface of macrophages [24]. It has been reported that ABCA1 is required for the export of annexin I from cells [31]. Therefore, whether the presence or absence of ABCA1 in mouse macrophages had any effect on the display of annexin I and II on the macrophage surface was examined. As shown in Figure 11, both annexin I and annexin II can be readily detected by monoclonal antibodies on the surface of elicited peritoneal macrophages. Their expression on cells from *ABCA1^−/−^* mice (Fig 11B) was comparable to expression on wildtype cells (Fig 11A), indicating that the presence of annexins I or II on the macrophage surface is not dependent on ABCA1.

## Discussion

A major advance in our understanding of cholesterol transport was the discovery that a defect in ABCA1 blocks the efflux of cholesterol and phospholipids from cells to apoA1 to form circulating HDL [1], [2], [32]–[34]. By itself, this discovery did not identify the mechanism by which the ABCA1 protein facilitates transfer of cholesterol to HDL, but good evidence suggests that it acts upstream of actual transfer of cholesterol from the cell. One proposal is that ABCA1 serves as a site for apoA1 to bind or dock on the cell surface [28], [35]. However, ABCA1 also facililitates cholesterol transfer to artificial amphipathic helical peptides, even those comprised of D-amino acids [36]; if providing a binding site is the important function of ABCA1, there cannot be stereospecificity for the ligands bound. Moreover, this proposal by itself fails to explain why the ATPase activity of the protein is required for ABCA1 function in the pathway [37]

Another proposal consistent with the involvement of ABCA1 early in the pathway forming HDL is that ABCA1 facilitates phospholipid, rather than cholesterol, transfer to newly synthesized, lipid-poor apoA1 to form a nascent HDL complex capable of accepting cholesterol [38]–[40]. Once this phospholipid transfer step has been completed, phospholipid-containing apoA1 particles would then accept cholesterol from cells without any further intervention from ABCA1. This proposal implies that ABCA1 interacts in some way with phospholipids, an attractive idea because it might explain how ABCA1 could be required for another function, also dependent on phospholipid interaction, namely the engulfment of apoptotic cells. In particular, ABCA1 has been proposed as the mammalian functional homolog of ced-7, an ABC protein required for clearance of apoptotic cells in *C. elegans*. The requirement for ced-7 function in both apoptotic target and phagocyte in nematodes [12] and the requirement for PS exposure in both target and phagocyte in mammals [17], [18], [20] suggests that the ced-7 homolog, ABCA1, might somehow contribute to the transbilayer phospholipid movements that result in PS exposure.

An understanding of the activities that control the transbilayer distribution of PS is required to properly evaluate whether ABCA1 might be involved. Ordinarily, PS is confined to the inner leaflet of mammalian plasma membranes by ATP-dependent inward transport catalyzed by a subfamily IV P-type ATPase, the aminophospholipid translocase [13], [14]. It is conceivable that apoA1 loading, like recognition of apoptotic cells, is sensitive to this lipid arrangement, although perhaps not to the resulting appearance of PS on the surface [41] . ABCA1 might modulate this arrangement directly by regulating the activity of the aminophospholipid translocase or indirectly, for instance, by ensuring proper localization of the translocase at the plasma membrane. However, the results presented in Figures 2 and 7 rule out these possibilities by showing that the presence or absence of ABCA1 has no effect on aminophospholipid transport activity.

PS comes to the cell surface in several physiological situations in addition to apoptosis, including upon platelet activation [42] and prior to myoblast fusion [43]. In apoptotic cells and platelets, the appearance of PS on the cell surface is a consequence of two events [15], [44]. As might be expected, one is the downregulation of the aminophospholipid translocase. The other is the upregulation of the scramblase, which facilitates transbilayer movements of PS to the cell surface. The properties of the scramblase in both cell types is the same (Fig 1): all types of phospholipids are moved at identical rates in both directions across the bilayer [21], [45]. The studies with ABCA1-deficient mouse and human cells presented here collectively demonstrate that ABCA1 is not the (long-sought) scramblase activity itself in lymphocytes, fibroblasts, HeLa cells, or macrophages.

If not the scramblase itself, ABCA1 could still be involved in activation of the scramblase. Studies of cells from patients with a rare clotting disorder, Scott syndrome, suggest that two different activation pathways (Ca^2+^-dependent and apoptotic, Ca^2+^-independent) converge on a common scramblase enzyme. As summarized in Figure 1, ABCA1 could be a protein involved in one of these two activation pathways. In platelets and other hematopoietic cells, including lymphocytes, scramblase can be activated naturally or artificially by a Ca^2+^-dependent pathway that is disrupted by the Scott mutation. It was recently reported that there is a single missense mutation in the ABCA1 gene of a Scott patient and that transfection of wild-type ABCA1 into lymphocytes from the patient restored Ca^2+^-dependent activation of the scramblase [46]. These results suggested that reduced ABCA1 activity might result in a failure of PS exposure like that observed in the Scott syndrome. As shown here, however, this inference is incorrect: PS exposure by the scramblase is activated by Ca^2+^ equally efficiently whether or not cells contain a functional ABCA1 gene (Figures 3 and 9). Similarly, it has been reported that scramblase activation by ionophore and Ca^2+^ is negligible in fibroblasts from *ABCA1^−/−^* mice [7] As confirmed here, human fibroblasts from Tangier patients do not activate the scramblase upon treatment with ionophore and Ca^2+^; however, this phenotype is also observed in wild type fibroblasts, suggesting that the ability to activate the scramblase by elevation of cytoplasmic Ca^2+^ may only be prominent in the hematopoietic lineage. In apoptotic cells, including lymphocytes, scramblase is activated by a Ca^2+^-independent mechanism which is unaffected by the Scott mutation. As shown in Figure 4, the absence of ABCA1 does not affect PS exposure via this apoptotic activation pathway, either.

A third possibility for involvement of ABCA1 in PS exposure, shown in Figure 1, is that ABCA1, independent of the scramblase, actively transports PS, and not other lipids, from the inner to the outer leaflet of the plasma membrane. An activity of this sort was first proposed to explain PS exposure in activated platelets [47], but high resolution analysis of lipid movements in activated platelets failed to reveal any evidence for PS-specific externalization over and above the scramblase-mediated movement [45]. More recently, analyses of movements of spin-labeled PC and PS analogs were also interpreted as evidence for an outward PS-specific transport in mouse red blood cells treated with Ca^2+^ and ionophore [7], conditions known to activate the scramblase in human red blood cells [48]. In these experiments, the induction of rapid internalization of PC, characteristic of the scramblase activity, was unaffected by knockout of the ABCA1 gene. The behavior of the PS probe, however, was more complex. In red blood cells from wild type mice, externalization of labeled PS was slow and only 24% of it moved from the inner to the outer membrane leaflet. In *ABCA1^−/−^* mice, externalization was similarly slow, and only 10% of internalized probe was externalized. On the basis of these findings, the investigators concluded that Apart of PS externalization in the presence of cytosolic Ca^2+^ may be associated with ABCA1-dependent selective outward transport@. This model suggests that PS externalization results from both non-specific scramblase and PS-specific ABCA1 activity, and that when the latter is compromised, the rate of externalization of PS should be reduced to the rate of scramblase-mediated movement alone.

Although suggestive, these data are difficult to reconcile with previous studies of lipid movements in ionophore treated human red blood cells [48]. In these earlier studies with fluorescent probes, scramblase activation resulted in PS movement as rapid, and as extensive, as PC movement. Similar results were obtained in studies of the scramblase activity in platelets [45] and in lymphoid cells [22] using both fluorescent phospholipid analogs and continuous annexin V binding assays of endogenous PS movement. The failure of Hamon et al to observe PS equilibration in normal controls makes it difficult to interpret their findings in ABCA1^−/−^ cells. The measurements of PS externalization rates reported here fail to confirm that ABCA1 deletion reduces the rate of PS externalization. Because ABCA1 ablation has no effect on the PS externalization rate in lymphocytes or macrophages activated by Ca^2+^ and ionophore, it is unlikely that ABCA1 catalyzes PS-specific externalization depicted as a possibility in Figure 1.

These findings are reassuring in one way, because an activity that actively exports one major phospholipid population from one leaflet to the other, when activated, would pose a difficult problem for the maintenance of membrane integrity. Although PS comprises only approximately 10% of plasma membrane phospholipid, even a simple equilibration of only this lipid between the two leaflets (from 100% inner to 50/50, inner/outer) would produce a 10% difference in the surface areas of the two leaflets, an unlikely event given that membranes cannot tolerate a difference of more than 1–2% in the surface area of the two leaflets. Active transport of all PS to the cell surface would be twice as disruptive. Indeed, this potentially disruptive effect of lipid movement may explain why the scramblase activity is non-specific for phospholipid headgroup. Although its function may be to bring PS to the cell surface, its nonspecificity provides an automatic compensation mechanism which dissipates any disparity in leaflet surface area as fast as it arises.

Because ABCA1 is prominently expressed in macrophages, it might seem likely, *a priori*, that the characteristics and function of this cell type would be affected by ABCA1 inactivation. The elevated level of annexin V binding to normal macrophages suggests that transbilayer lipid movements are altered in these cells, and it has been reported that annexin V binding to macrophages from ABCA1^−/−^ mice is reduced [7], although the data were not presented. The development of methods to measure lipid movements in macrophages reported here provides the first opportunity to actually examine this dynamic phenomenon in these membrane-active cells, rather than to measure just static levels of exposed PS using annexin V. The results presented here provide evidence that many of the transbilayer lipid movements seen in other hematopoietic cells are present in macrophages as well, including both translocase and Ca^2+^-induced scramblase activity. However, the assays also indicate that none of these transbilayer lipid movements, nor the elevated level of basal PS exposure measured by annexin V binding, requires the presence of ABCA1 in macrophages (Figures 6–9). In addition, a reduction in Ca^2+^-dependent annexin V binding by the F4/80-positive cells in the cell population could not be detected. These results indicate that ABCA1 is not responsible for readily-measurable lipid transport activity in macrophages, including catalysis of PS-specific externalization. In keeping with these observations, elicited peritoneal macrophages from ABCA1^−/−^ mice were no less effective than wild type cells as phagocytes of apoptotic thymocytes in a controlled, *in vitro* assay. Although these results do not rule out a role for ABCA1 in the recognition of apoptotic cells, they do indicate that the contribution of ABCA1 to engulfment is quantitatively much less significant than that of the many other molecules identified by these *in vitro* assays, such as CD14 [49], [50], integrins [30], [51], or annexin I [24].

A potential positive effect of ABCA1 activity was suggested by reports that introduction of ABCA1 into HeLa cells specifically activated spontaneous externalization of previously internalized NBD-PS [52] In the higher resolution experiments described here with an independent line of ABCA1-transfected HeLa cells, no difference in externalization was observed. In addition, no reduction in the rate of basal PS efflux was observed in primary fibroblasts, macrophages, or lymphocytes lacking the ABCA1^−/− ^gene. Some experiments have suggested that ABCA1 is essential for loading of phospholipid onto apoA1 to convert it to a form capable of taking up cholesterol [40], [53]. The experiments shown here demonstrate that the function provided by ABCA1 is not PS externalization stimulated by the presence of apoA1, at least on any large scale.

Understanding the function of ABCA1 in the efflux of cholesterol to HDL is also a persistent puzzle. The possibility that a phospholipid transport function was a key to solving this puzzle was a welcome unifying idea, because of the clear and defined importance of phospholipid transport in the process of clearance of apoptotic cells. The molecules that are responsible for mediating and regulating the transbilayer transport of PS required for recognition of apoptotic cells (among other functions) have resisted identification, and the possibility that ABCA1 is one of those molecules was an important potential implication of this unifying idea. The results presented here indicate that this possibility is not realized-ABCA1 plays no readily identifiable role as translocase, scramblase, or regulator of either of those molecules in either the Ca^2+^-stimulated or apoptosis-stimulated pathways in hematopoietic or fibroblast cells. These results do not rule out a role for ABCA1 in phospholipid loading of apoA1. Indeed, recent studies suggest that this enzyme plays a role in changing the conformation of apoA1, and loading as many as two PC molecules on the rearranged protein [40]. The loading of PC molecules in those studies is unlikely to involve transbilayer lipid movement, since PC is normally concentrated in the external leaflet of the plasma membrane, and the number of PC molecules loaded is small compared to the number of PC molecules present in the outer leaflet. Whether ABCA1 might function in a similar fashion during the process of apoptotic cell recognition will be an interesting possibility for future consideration.

## Materials and Methods

### Reagents

ApoA-1(A-0722), propidium iodide (PI), RPMI-1640 and α-MEM medium, fatty acid free bovine serum albumin (BSA, A-6003), dexamethasone, purified mouse IgG1 (clone MOPC 21) mAb isotype control, fluorescein isothiocyanate (FITC)-conjugated goat anti-mouse IgG and unconjugated goat IgG were purchased from Sigma-Aldrich (St. Louis, MO). Glibenclamide/Glyburide was from Research Biochemicals International (Natick, MA). Camptothecin was from MP Biomedicals (Irvine, CA). Ca^2+^ ionophore A23187 was from Calbiochem (EMD Biosciences, Inc., San Diego, CA). Diff-Quik staining reagents were from Baxter (Miami, FL). Hygromycin B, geneticin selective antibiotic (G418) and trypsin-EDTA (0.25% trypsin, 1mM EDTA) were from Gibco (InVitrogen, Carlsbad, CA). FITC-conjugated rat anti-mouse F4/80 mAb (clone CI:A3-1, IgG2b) and FITC-conjugated rat mAb IgG2b (clone LO-DNP-11) isotype control were purchased from Serotec (Raleigh, NC). Mouse anti-annexin I mAb (clone 29, isotype IgG1) generated against bovine annexin I, and mouse anti-annexin II mAb (clone 5, isotype IgG1) generated against bovine annexin II were purchased from BD Transduction Laboratories (Lexington, KY). 1-oleoyl-2-[6[(7-nitro-2,1,3-benzoxadiazol-4-yl)amino] caproyl]-sn-glycero-3-phosphocholine (NBD-PC) and the corresponding phosphatidylserine analog (NBD-PS) were purchased from Avanti Polar Lipids (Alabaster, AL). Fluoresceinated annexin V was either FITC-conjugated annexin V purchased from Molecular Probes (Eugene, OR) or 5,6-carboxyfluorescein (5,6-FAM)-derivatized recombinant annexin V (see below).

### Preparation of fluorescein-derivatized annexin V

4 mg of recombinant human annexin V, purified as described previously [20], in 2 mL of Tris buffer was dialyzed overnight at 4°C against 200 mM sodium bicarbonate, pH 8.5, to which 0.2 mg of 5,6 FAM-succinimide (AnaSpec, Inc., San Jose, CA) was then added from a 10 mg/ml stock in DMF. After mixing for 1 hr at 25°C, protected from light, the reaction mixture was dialyzed extensively in the dark against 25 mM HEPES, pH 7.4, 140 mM NaCl, 1 mM EDTA before dilution to 45 μg of protein/ml. Bovine serum albumin (BSA) and sodium azide were added to 10 mg/ml and 0.01%, respectively.

### Cell culture

HeLa (Tet-off) cells obtained from Clontech (Mt. View, CA) and co-transfected with pTRE2-ABCA1-GFP and pTK-Hyg (to provide a selectable marker) or transfected with pTK-Hyg alone (control) , human EBV-transformed B lymphocyte lines derived from a normal individual (ARB) and a Tangier disease patient (ARA), and normal human fibroblasts (line 1947) and human fibroblasts derived from a Tangier disease patient have been previously described [25]. ABCA1 genotypes of EBV-transformed B lymphocytes were verified by standard PCR [32]. HeLa cells were maintained in α-MEM medium supplemented with 10% fetal bovine serum (FBS), 50 U/ml of penicillin, 50 μg/ml of streptomycin, 200 μM hygromycin B, and 200 μM G418, at 37°C and 5% CO_2_. EBV-transformed B lymphocyte lines were maintained in RPMI 1640 medium plus 10% FBS , 50 U/ml of penicillin, and 50 μg/ml of streptomycin at 37°C and 5% CO_2_. Fibroblast cell lines were grown in α-MEM medium with the same additives at 37°C and 5% CO_2_ and were maintained at a low passage number. For cell passage and analysis in suspension, cells were removed from monolayer culture by a 30 sec exposure to trypsin-EDTA. Jurkat cells and the J774A.1 mouse macrophage cell line (American Type Culture Collection) were maintained in RPMI 1640 medium supplemented with 10% FBS, 100 U/ml of penicillin and 100 μg/ml of streptomycin at 37°C and 5% CO_2_.

### ABCA1 null mutant mice

A heterozygous pair of strain DBA/1-*Abca1^tm1Jdm^* (stock number 003897) mice was purchased from the Jackson Laboratory (Bar Harbor, ME) and maintained on regular chow and water *ad libitum*, in accordance with the institutional guidelines of the Animal Care and Use Committee. A breeding colony derived from this original pair was the source of ABCA1 homozygous (−/−), heterozygous (+/−), and wildtype (+/+) mice used in this study. Mice were genotyped as described (http://jaxmice.jax.org/pub-cgi/protocols/protocols.sh?objtype = protocol&protocol_id = 465).

### Primary mouse macrophages

Inflammatory macrophages were elicited in the peritoneal cavity of 6–8 wk old mice by intraperitoneal injection of 2 ml of 3% Brewer = s thioglycollate. Cells were harvested 5–6 days later by peritoneal lavage using 10 ml of ice-cold RPMI 1640 medium. The collected cells were washed in RPMI 1640 medium and suspended at a concentration of 3×10^6^ cells/ml in RPMI 1640 medium containing 10% FBS. For enrichment of macrophages for phagocytosis assays, cell suspensions (150 μL) were pipetted onto 18 mm bicarbonate-treated glass coverslips placed in the wells of a 12-well culture plate. After 2 h at 37°C and 5% CO_2_, nonadherent cells were removed by aspiration. Then, 1 ml of fresh RPMI 1640 medium containing 10% FBS was added to each well and cultures were incubated overnight at 37°C and 5% CO_2_ before phagocytosis assays.

### Apoptotic targets

Thymuses were removed from 6–8 wk-old mice and dissociated in RPMI 1640 medium. Following centrifugation, cells were resuspended at 4–5×10^6^ cells/ml in RPMI 1640 medium. To produce apoptotic targets, 10^−6^ M dexamethasone was added and cells were incubated for 4–6 h at 37°C and 5% CO_2_. EBV-transformed B lymphocyte lines, maintained in RPMI 1640 medium plus 10% FBS, were treated with 4μg/ml of camptothecin for 6 h to induce apoptosis.

### mAb staining

10^6^ cells from peritoneal lavages were preincubated in 50 uL of PBS containing 1% FBS and goat IgG at 1.4 mg/ml for 5 min on ice. FITC-anti mouse F4/80 mAb or FITC-IgG_2b_ isotype control mAb was then added to the suspension at a final concentration of 10 μg/ml. Staining with anti-annexin I, anti-annexin II, and IgG_1_ isotype control antibodies was as previously described [24].

### Annexin V staining

FITC- or 5,6 FAM-annexin V (0.5 uL) was added to 2×10^5^ EBV-transformed B lymphocytes or 5×10^5^ peritoneal lavage cells in 50 μL of annexin V binding buffer (ABB,10 mM HEPES, pH 7.4, 140 mM NaCl, 2 mM CaCl_2_) or ABB without Ca^2+^. After 5 min on ice, the volume was brought to 500 uL by addition of the same buffer. PI was added to a final concentration of 10 ug/ml immediately before flow cytometric analysis.

### Flow cytometry

Cells (10^4^) were analyzed at room temperature using an EPICS-XL-MCL flow cytometer (Coulter Electronics, Hialeah, FL) with excitation at 488 nm. FITC (or FAM) staining was monitored at 525 nm. PI fluorescence was monitored at 610 nm and PI-positive cells were gated out of all profiles. Forward and side light scatter properties were used to gate for morphologically normal lymphocytes, macrophages and fibroblasts, which include both non-apoptotic and early apoptotic cells. All experiments were repeated at least once, and, in most cases, many times. For comparisons, samples of different genotype were always examined in parallel, using the same reagents and cytometer settings, to eliminate differences resulting from day-to-day variations in instrument performance, reagent stability, etc.

### Phagocytosis assay

All assays were performed in triplicate. Thymocytes or camptothecin-treated EBV-transformed B lymphocytes (1.5×10^6^) in 150 μL of ABB were overlaid onto cultures of either elicited peritoneal macrophages or J774.1 cells prepared and washed as previously described [20]. In some experiments, EBV-transformed B lymphocytes were incubated for 15 min in 10 µM annexin V in ABB prior to washing and resuspending in 150 μL ABB and presentation to macrophages as described above. After 30 min at 37°C and 5% CO_2_, coverslips were washed vigorously with phosphate buffered saline (PBS), fixed in 1.8% formaldehyde for 5 min, and then submerged in PBS until counted. Just prior to counting, fixed cells on coverslips were stained with Diff-Quik and then phagocytosed cells enumerated as described in detail previously [30]

### Continuous annexin V binding assay for PS externalization

Briefly, 10^5^ cells were suspended in 750 μL of ABB containing 0.7 μg/ml of PI and 1-2 μL of FITC- or FAM-annexin V. Ca^2+^ ionophore A23187 was added to a final concentration of 2-7 μM and the cell suspension was then sampled continuously in the flow cytometer, as previously described [22].

### NBD-phospholipid translocase assays

10^5^ HeLa cells, fibroblasts or B lymphocytes, washed free of serum, were resuspended in 800 μL of HEPES buffer (10 mM HEPES, pH 7.4, 150 mM NaCl) and PI was added to a final concentration of 1.2 μg/ml. 6 μL of NBD-PS or NBD-PC, dried from a concentrated chloroform stock and resuspended in HEPES buffer to 30 μM, was then added, and 50 μL samples were taken at indicated time intervals and diluted into 200 μL of HEPES buffer (pre-cooled on ice) with 1% fatty acid-free BSA, to extract label remaining in the outer leaflet of the membrane, or without BSA, to measure total fluorescence. After 2 min for extraction, samples were introduced into the flow cytometer and fluorescence measured as previously described [22]. To reduce noise introduced by the presence of small numbers of very bright cells in the population, median fluorescence was used as the measure of population fluorescence intensities. For mouse macrophages, 10^5^ peritoneal exudate cells were resuspended in 600 μL of ABB and PI was added as above. NBD-PS or NBD-PC was added as described above but in order to ameliorate the effects of probe uptake by endocytosis, samples (50 μL) were added to 200 μL of Hanks buffer, and 2.5 μL of a freshly prepared 1.0 M stock solution of sodium dithionite in 1.0 M Tris-HCl was added 5 min before measurement by flow cytometry as described [22].

### NBD-PS externalization assay

A continuous flow cytometric assay, similar to a spectro-fluorometric assay described previously for platelets [45]and cultured cells [22], was used to monitor the outward movement of NBD-PS. Cells (10^5^), suspended in 750 μL of ABB or medium without serum, were first preloaded with NBD-PS by adding 2 μL (for HeLa , macrophages and lymphocytes) or 10μL (for fibroblasts) of a 30 μM stock of NBD-PS (prepared as described above) followed by incubation for 3 min, to minimize the level of probe internalized by nonspecific endocytosis (macrophages) or 10 min (lymphocytes, Hela and fibroblasts). A 10 μL aliquot of a 1M solution of sodium dithionite (freshly prepared as described above) was then added and the cell suspension was analyzed continuously by flow cytometry for 15 min at room temperature to measure the fraction of internalized NBD-PS remaining inside the cell. For measurements of the effect of apoA-1 on PS externalization in B lymphocytes and fibroblasts, 10 μg/ml of apoA-1 was added and incubation was continued for 5 min, prior to the addition of sodium dithionite. Measured rates were routinely tested for sensitivity to the concentration of added dithionite as a control for membrane permeability to dithionite ions.
